# ^18^F-FDG PET/CT in lymphangiosarcoma: A case report and review of literature

**DOI:** 10.22038/aojnmb.2024.77689.1548

**Published:** 2025

**Authors:** Nitin Gupta

**Affiliations:** Department of Nuclear medicine, Dr Rajendra Prasad government medical college, India

**Keywords:** Breast carcinoma, FDG PET/CT Lymphagiosarcoma, Stewart-Treves Syndrome

## Abstract

**Objective(s):**

Lymphangiosarcoma is a rare tumor that affects the upper limbs of patients who have undergone breast cancer surgery, including axillary dissection, followed by radiation therapy (RT) to the axilla and has a poor prognosis. ^18^F-FDG PET/CT may enable the earlier detection of malignant transformation in a setting of chronic lymphedema and help evaluate the extent and staging of the tumor, allowing earlier initiation of treatment options.

**Case presentation:**

We herein report a case of cutaneous lymphangiosarcoma in a 47-year-old breast carcinoma patient, which occurred 9 years after initial surgery and radiation therapy. Distant metastases were detected on ^18^F-FDG PET/CT. The patient underwent fore-quarter amputation of the upper limb and concurrent chemo-radiation therapy. However, she succumbed to her disease after 3 cycles of chemotherapy.

**Conclusions:**

^18^F-FDG PET/CT scan helps in the early detection of malignant transformation and lymphangiosarcoma in a setting of chronic lymphedema in breast carcinoma patients following radiation therapy to the axilla. Furthermore, it helps determine the extent of regional spread and detect metastatic involvement, thus enabling better clinical management of these patients.

## Introduction

 Cutaneous lymphangiosarcomas account for approximately 5% of all angiosarcomas and usually originate in limbs with chronic lymphedema. Also known as Stewart-Treves Syndrome (STS), it is a very rare skin angiosarcoma with a very poor prognosis. It typically affects the upper limbs of patients who have undergone breast cancer surgery, including axillary dissection, followed by radiation therapy (1, 2). Its incidence is reported to be between 0.07% and 0.45% in breast cancer patients surviving at least 5 years after radical mastectomy (3). 

 Regarding its etiopathogenesis, radiation therapy to the axilla leads to the irradiation of axillary lymph nodes, causing their sclerosis, which results in lymphatic blockade manifesting as chronic lymphedema (4). 

 Magnetic resonance imaging (MRI) is the examination of choice for regional characterization and local staging of sarcomas (5). F-18 Fluorodeoxyglucose positron emission tomography/computed tomography (^18^F-FDG PET/CT) has been proposed as an important tool in staging and treatment planning of STS (6). In this article, we present a case report of a patient diagnosed with STS at our department, together with a literature review.

## Case presentation

 A 47-year-old female patient had undergone left-sided modified radical mastectomy and axillary dissection in September 2014 with ﬁndings of moderately differentiated intraductal carcinoma of left breast - pT2N3bM0, positive resected nodes 11/26, and negative ER, PR, and Her2 Nu. 

 Between November and January 2014, she had received 6 cycles of adjuvant chemotherapy with Docetaxel, Doxorubicin, and Cyclo-phosphamide. Subsequently, in February and March 2015, she had undergone adjuvant radiation therapy to the left chest wall and axilla. Thereafter, breast cancer remained in complete remission throughout the period of follow-up.

 However, about 18 months after the surgery and RT, she developed lymphedema of the left upper limb. She had been recommended limb elevation and physiotherapy and to wear a compression sleeve. However, despite these measures, the edema persisted and continued to progress slowly and gradually to the distal forearm, wrist, and hand.

 In January 2023, the patient noticed the appearance of some maculopapular rash-like lesions along the anteromedial aspect of the edematous upper arm, some of which had ulcerated with purulent discharge, with redness and pain in the affected area. She was treated at a local clinic. The lesion was suspected as cellulitis, and a culture of the discharge was done, which showed Streptococcus pyogenes. 

 Subsequently, she was treated with systemic and local antibiotics over 2 weeks, and the purulent discharge was resolved. However, the lesions still persisted, and she was again given an antibiotic course.

 Eventually, the patient came to our department 3 months after receiving initial treatment from the local clinic. She complained of progression in the size of lesions with the appearance of nodular lesions and ulceration and marked induration of the involved area. On examination, she was afebrile and presented with lymphedema of the entire left arm. On local examination, there was an irregular nodular ulceroproliferative lesion ~6×2cm with erythema and induration in the anterior aspect of the left upper arm. The lesion was firm to hard in consistency and appeared to be fixed to thickened underlying skin.

 Due to the patient's prolonged history of lymphedema, the clinical appearance of the lesion, and the lack of improvement with antibiotic treatments, there was a suspicion of a malignant tumor. Differential diagnoses considered included cutaneous metastasis from breast cancer, lymphangiosarcoma, basal cell carcinoma, squamous cell carcinoma, and malignant melanoma.

 Magnetic resonance imaging was performed for characterization and assessment of the extent of the lesion. The T1-weighted MRI showed an ulcerated hypointense cutaneous lesion ~7.5×2.9 cm with few other sub centimetric soft tissue satellite lesions in the cutaneous and subcutaneous tissue of the left arm, which were hyperintense on T2-weighted images and showed marked contrast enhancement. No other mass lesions were seen. 

**Figure 1.1:**
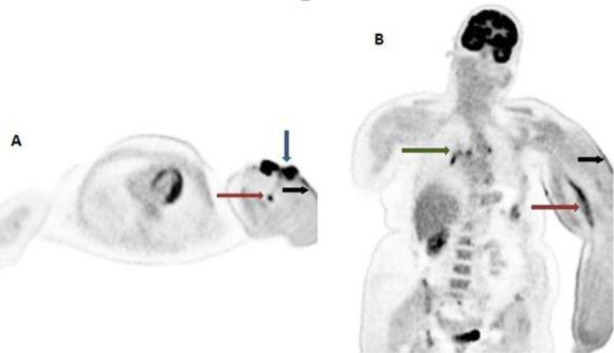
47 year-old female patient post mastectomy, axillary dissection and radiotherapy to left axilla had developed gradually increasing lymphedema of the left arm 18 months post radiotherapy. The patient complained of muculopapular rash like lesion in the edematous arm approximately seven years after the onset of edema, which later progressed to nodular ulcerated lesion. FDG PET MIP images; (**A**. axial) show nodular foci of uptake along anterolateral aspect of left arm (**blue arrow**), and in the region of left humeral shaft (**red arrow**). **B**. Coronal PET images show linear FDG uptake in the left arm (**red arrow**) and few foci of FDG upake in the mediatinal region (**green arrow**). The left arm appears edematous with cutaneous thickening (**black arrow**)

 The patient was then referred for an ^18^F-FDG PET/CT scan for disease extent and metastatic workup, which showed a high-grade FDG avid (SUV_max_=20.8) irregular ulceroproliferative lesion ~7.4×2.8 cm involving the cutaneous and subcutaneous tissue of the left arm, with few other discrete infiltrative satellite tiny nodular lesions and marked stranding of the subcutaneous tissue. In addition, an ill-defined soft tissue nodular lesion (SUV_max_=6.3) was seen abutting the cortex of the humeral shaft with moderate grade FDG uptake along the cortex of the adjacent humeral shaft without any discernible cortical erosion. There were also identified FDG-avid lymph nodes in the right supraclavicular and mediastinal regions (SUV_max_=8.8~1.1×1.0 cm; subcarinal) (Figures 1.1, a.2 and 1.3).

**Figure 1.2:**
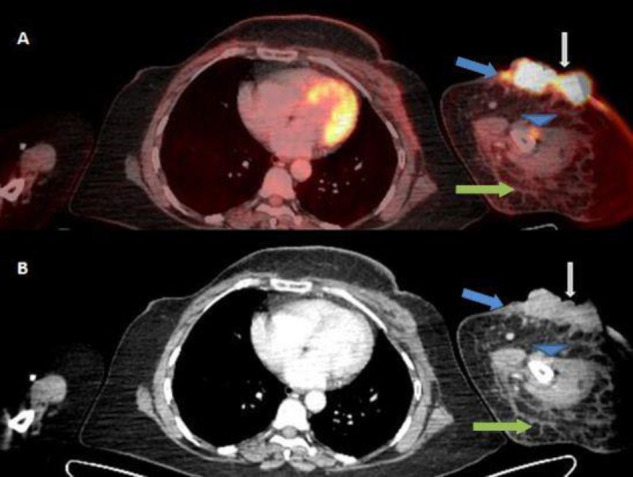
Corresponding fused axial **A.** FDG PET/CT and **B.** CECT images show FDG avid (SUV_max_ 20.8) heterogenous enhancing slightly exophytic cutaneous lesion in antero lateral aspect of left arm ( **white arrow**), with adjacent cutaneous thickening( SUV_max_ 7.8) ( **blue arrow**), edema and stranding of the subcutaneous fat (**green** **arrow**) and a subcentimetric nodular soft tissue ( SUV_max_ 6.3) abutting the shaft the humerus ( **blue arrow head**)

**Figure 1.3:**
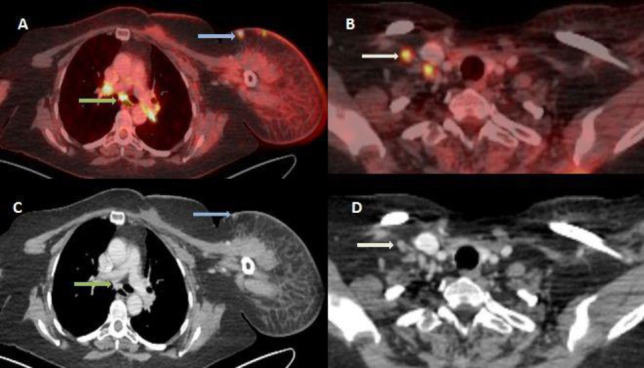
Fused axial FDG PET/CT (**A** and **B**) and corresponding CECT image (**C **and **D**) show FDG avid bilateral hilar and subcarinal (SUV_max_ 8.8) lymph nodes (**yellow arrow**), right supraclavicular lymph nodes (SUV_max_ 7.4; **white arrow) **and few subcentimetric subcutaneous nodules (SUV_max_ 5.1; **light blue arrow**).

 A biopsy of the lesion was performed, and histological examination (Figure 2.1) showed vascular breakthroughs in the dermis, with round to oval-shaped cells with hyperchromatic and pleomorphic nuclei, large nucleoli, and mitotic figures. Variable-sized lumens were also observed throughout the tumor. 

 Immunohistochemical studies (Figure 2.2) revealed that the tumor cells were positive for CD31, FLI-1, D2-40, Prox-1, and Myc amplification and negative for Cytokeratin, epithelial membrane antigen, CD20, and CD34. She also underwent an excision biopsy of right supraclavicular lymph nodes, which were found to be metastatic lesions. Based on clinical history, histopathology, and immuno-histochemistry, she was diagnosed with lymphangiosarcoma with metastatic right supraclavicular and mediastinal lymph nodes. 

 Subsequently, she was planned and started on palliative chemotherapy with Paclitaxel (80 mg/m^2^ i.v. on days 1, 8, and 15). However, she succumbed to her disease after 3 cycles of chemotherapy.

**Figure 2 .1:**
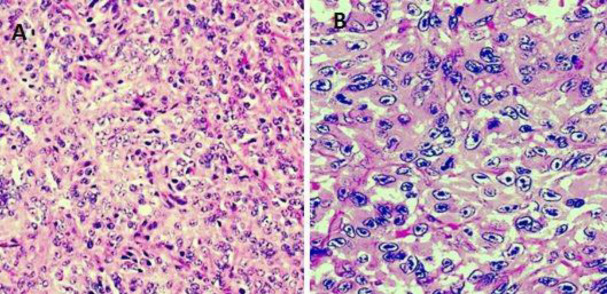
(**A** and **B**). The lesion showed sheets of spindle cells highly pleomorphic and hyperchromatic with interspersed mitotic figures (H and E, 100 X and 400 x respectively)

**Figure 2.2:**
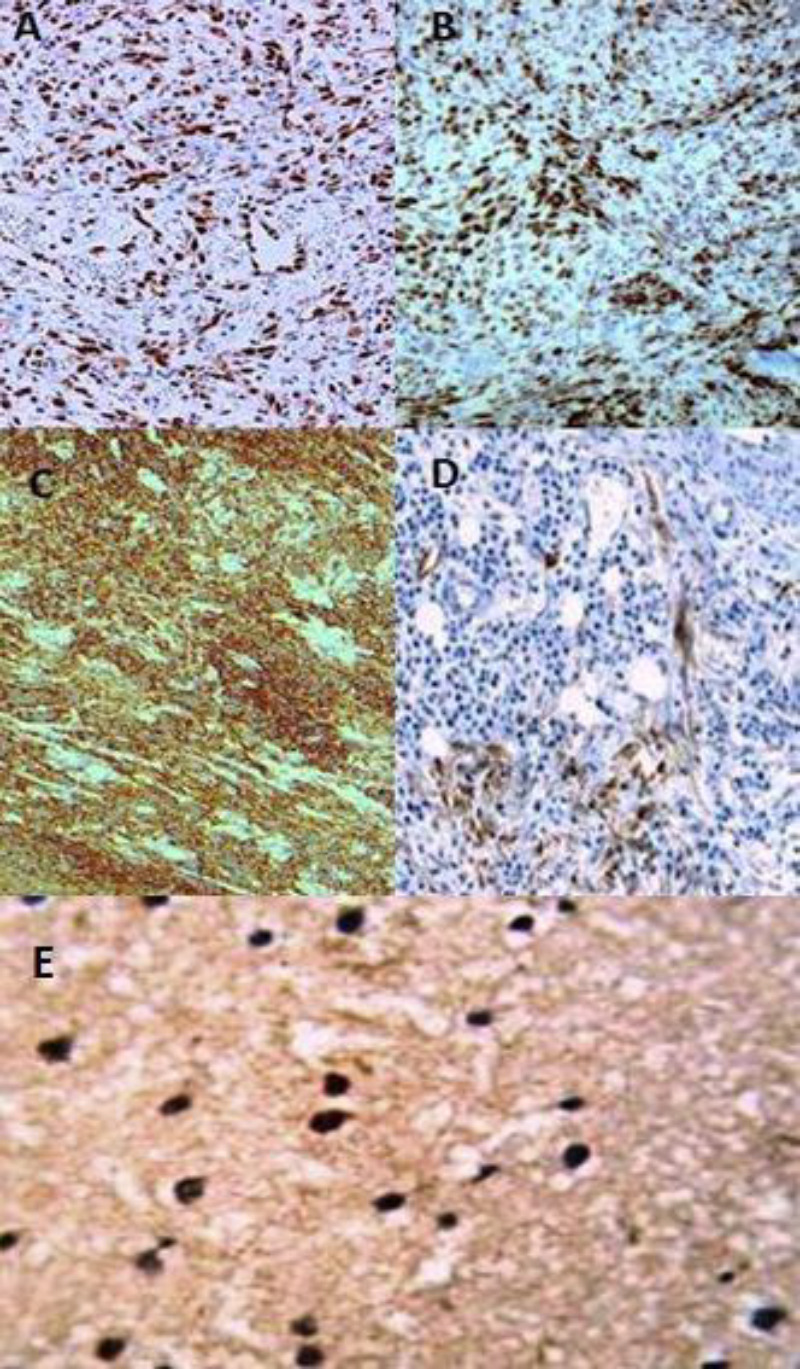
IHC of the tumor cells showing positive staining for **A**. FLI 1, **B**.myc, **C**. CD31 **D.** D2-40 and **E**. PROX1.

***Discussion and review of literature***

 Angiosarcomas account for less than 2% of all sarcomas in humans (7, 8). Cutaneous lymphangiosarcomas account for approximately 5% of all angiosarcomas and usually originate in a limb with chronic lymphedema. Lymphatic blockade, leading to chronic lymphedema, is the primary event in the onset of STS (9). Radiation therapy to the axilla causes axillary node sclerosis, resulting in lymphatic blockade and lymphedema (5). 

 Protein-rich interstitial ﬂuid alters local immune response in the affected limb, and the lymphatic channels enriched with growth factors, such as vascular endothelial growth factor (VEGF) (1), stimulate lymphangiogenesis and lead to the development of collateral vessels (4). Regional immunodeﬁciency results in the failure of immune surveillance mechanisms, and the lymphedemaous region becomes immunologically susceptible to malignancies, such as vascular tumors (10, 11).

 Lymphangiosarcoma usually manifests as fast-growing multiple red-blue macules with surrounding indurations, turning into plaques of coalescing papules with necrosis (1). 

 Lymphedema of the upper limb, which precedes cutaneous lesions, may be progressive and gradually spread from the arm to the forearm and dorsal part of the hand (12). The most common sites of possible metastatic spread are the lungs and thoracic wall, followed by the liver, bones, soft tissues, and lymph nodes (4, 13, 14).

 It is necessary to consider differential diagnosis from benign conditions, such as acquired angioedema, pyogenic granuloma, lymphangiectasis, angioendotheliomatosis, benign lymphangioendothelioma, lymph-angioma, and cellulitis, to malignant diseases, such as Kaposi sarcoma, malignant melanoma, lymphoma, squamous cell carcinoma, basal cell carcinoma, or skin metastases from other tumors (usually breast cancer) (1, 4, 14). Early diagnosis and radical surgical treatment are associated with a better prognosis (2). Thus, pre-treatment staging is paramount in choosing the correct treatment modality.

 Magnetic resonance imaging is the examination of choice for regional characterization and local staging of both bone and soft tissue tumors, including sarcomas (5). 

 Positron emission tomography is known to improve preoperative staging in patients with sarcoma (15, 16). It has been proposed as an important tool in staging and treatment planning of STS and has the potential to become the first-line choice in initial staging, assessing response to treatment and for follow-up because lymphangiosarcoma or STS has been shown to have a high ^18^F-FDG uptake (6, 17, 18). However, it is essential to be careful of the possible pitfalls in applying ^18^F-FDG PET/CT on lymphedema patients since ^18^F-FDG accumulates in all cells with a high glucose metabolism, such as in activated macrophages. 

 Hence, while FDG PET/CT has a high sensitivity, its specificity may be lower in cases where both infection and malignancies are present concomitantly (19). Therefore, histo-pathology and immuno-histochemical staining are essential for a definite diagnosis of lymph-angiosarcoma.

 Immuno-histochemical antibodies against endothelial cells are typically the stain of choice. 

However, not all malignant endothelial cells are positive for these markers; therefore, a panel of immuno-histochemical stains, including Von Willebrand Factor, CD31, Friend leukemia integration 1 transcription factor (FLI-1), D2-40, Myc amplification, Vascular endothelial growth factor receptor-3 (VEGFR-3), and Prospero homoebox-1 (Prox-1), should be used to avoid false negativity(20). 

 Only early radical surgical removal, including amputation or disarticulation of the affected limb or wide excision at an early stage, offers a chance of long-term survival (21). Systemic therapy with various cytostatics has been used in patients with advanced diseases. Some authors favor the use of Paclitaxel as the ﬁrst-line treatment for advanced or metastatic angiosarcoma (4, 13). 

 However, the overall prognosis of STS patients remains poor irrespective of the treatment modality chosen, with the overall survival ranging between 18 to 31 months (21). A high number of patients experience local and distant recurrences and die of metastatic disease within 2 years after the diagnosis (4, 23). 

 The interval for developing lymphangio-sarcoma in the patient presented by us was 9 years, which corresponds to published data (5, 24). 

 In the present case, ^18^F-FDG PET/CT demonstrated hypermetabolic activity in primary lesion cutaneous in the setting of chronic lymphedema, which, on biopsy and immuno-histochemistry, was diagnosed as lymphangiosarcoma. ^18^F-FDG PET/CT also detected adjacent hypermetabolic cutaneous thickening and a few hypermetabolic satellite nodules. In addition, ^18^F-FDG PET/CT also demonstrated hypermetabolic right supra-clavicular and mediastinal lymph nodes. 

 Excision biopsy and immuno-histochemistry of the right supra clavicular lymph node proved them to be metastatic lesions. Biopsy of mediastinal lymph nodes was not feasible; nevertheless, due to their morphological appearance on CT images and high metabolic activity, they were considered to be metastatic. 

 Thus, PET/CT not only helped characterize the primary lesion, demonstrating its malignant transformation, but it also showed the extent of the lesion and its metastatic spread. In a case report, Joshi et al. (25) described the FDG PET/CT and MRI appearance of STS lesions and the utility of ^18^F-FDG PET/CT for the characterization of the primary lesion and whole-body evaluation in these patients. 

 Another case report by Chen et al. (26) described the utility of ^18^F-FGD PET/CT in the early detection of malignant transformation and metastatic disease in patients with angiosarcoma arising in a setting of chronic lymphedema. In another case report, which included two patients, Dawlatly et al. (27) also described ^18^F-FDG PET/CT scan as extremely helpful in demonstrating the extent of subcutaneous spread and planning surgical management in such patients. These studies emphasize the importance of early characterization and accurate initial staging using whole-body ^18^F-FDG PET/CT to plan the appropriate treatment option by excluding metastatic involvement.

## Conclusions

 Though rare, lymphangiosarcoma should be considered a differential diagnosis in patients presenting with plaque such as coalescing maculopapular or ulcerative nodular lesions with a background of chronic lymphedema of the upper limb in breast carcinoma patients after radiation therapy to the axilla. ^18^F-FDG PET/CT plays an important role in the characterization of the primary lesion, enabling the early detection of malignant transformation. 

 It is also useful for demonstrating the extent of the spread of the disease and the detection of distant metastasis, thereby guiding optimum management of these patients, lowering the risk of recurrence, preventing the need for re-excision, and improving the overall prognosis.

## List of Abbreviations

^18^F-FDG: F18- Fluoro Deoxy Glucose

PET/CT: Positron Emission Tomography/ Computed Tomography

RT: Radiation Therapy

STS: Stewart-Treves Syndrome

H and E: Haematoxylin and Eosin
